# Occupational health professionals’ knowledge, understanding and use of work ability

**DOI:** 10.1093/occmed/kqt070

**Published:** 2013-06-14

**Authors:** K. Coomer, J. Houdmont

**Affiliations:** 1 ^1^KC Business Health Ltd, York YO31 1EY, UK,; 2 ^2^Occupational Health Psychology, Institute of Work, Health & Organisations, University of Nottingham, International House, Nottingham NG8 1BB, UK.

**Keywords:** Finland, occupational health nurses, occupational health physicians, United Kingdom, work ability, Work Ability Index

## Abstract

**Background:**

The concept of work ability (WA) has a 30-year history in Finland, where it has been used extensively in occupational health (OH) research and practice. The extent to which WA has been integrated into UK OH practice is unclear.

**Aims:**

(i) To compare knowledge, understanding and use of WA among OH nurses and physicians in the UK and Finland and (ii) to identify factors that influence the use of WA in Finnish OH practice.

**Methods:**

An online questionnaire administered to OH practitioners in the UK and Finland.

**Results:**

A total of 436 UK and 97 Finnish OH practitioners completed the questionnaire. Though familiarity with the term ‘work ability’ was similar among Finnish and UK respondents, substantial differences were found in understanding of the term. Ninety-five per cent (Finland) and 7% (UK) of respondents reported using the Work Ability Index (WAI), a validated measure of WA, in their practice. Finnish respondents indicated that they used the WAI results primarily for individual case management, understanding population health trends, health promotion and determining WA across age groups. UK respondents primarily attributed failure to use the WAI to lack of training. Primary factors influencing use of WA in Finland included it being considered common practice and an effect ive system by which to conduct individual assessments.

**Conclusions:**

There are large differences between Finland and the UK in the assessment of WA in OH practice. Differences may reflect contrasting OH legislative frameworks.

## Introduction

In recent years, the work ability (WA) construct has become an increasingly popular focus of attention in occupational health (OH) research [1,2]. WA and its associated measurement instrument, the Work Ability Index (WAI), have also been used extensively in OH practice in a number of countries [3–7], in addition to Finland, from where they originate [3,8]. Anecdotal evidence suggests that OH professionals in the UK have begun to use, or express interest in using, WA in response to its increasingly widespread use in OH research, the gradual accumulation of information from Finland on its potential uses in OH practice and the challenges presented to OH management by an ageing workforce. To date, there is no scientific evidence on the extent to which UK-based OH professionals are aware of, understand and consider WA in their practice. This study explores these issues among OH nurses and physicians from the UK and Finland. Comparisons are made to highlight possible areas for developing the use of WA in UK-based OH practice.

WA can be defined as a worker’s capacity to manage their job demands in relation to their health and mental resources. It takes into account all the factors that may influence that capacity and make the job more or less manageable [9]. Given that work demands are likely to change over the course of a career, the challenge over time is to balance demands and capacity in order to optimize the ability to work. Ilmarinen [10] describes the 30-year development of WA and the WAI in Finland in three key phases. The evolution stage (1980–89) involved the development of the WAI as a self-reported measure of seven dimensions of WA and functional capacity. During this period longitudinal WA research in Finland demonstrated WA decrements as workers age. The second stage, of conceptualization and implementation (1990–99), involved the training of Finnish OH physicians and nurses in the use of the WAI and the development of Finnish programmes promoting the use of WA in the workforce. The third stage (2000–09) involved translation of the WAI into 24 languages [8], presentations at numerous international conferences [11–13] and the development of further age management research activities across Europe.

In light of the challenges produced by an ageing workforce in the UK, and the Finnish and international evidence which demonstrates the utility of WA in OH practice, UK OH professionals might be expected to have embraced WA. However, the extent to which this is the case remains unclear, and a recent Google Scholar search, using the keyword ‘work ability index’, produced 1960 articles, of which as far as the authors are aware only three were UK specific [11,13,14].

Therefore, this study had two related aims:

(i) To compare knowledge, understanding and use of WA among OH practitioners in the United Kingdom and Finland.
(ii) To identify factors that influence use of the WAI in Finnish OH practice.

## Methods

A questionnaire to explore OH practitioners’ knowledge and understanding of WA and use of the WAI was developed, including questions such as ‘What do you understand by the term work ability?’, ‘Have you heard of the WAI?’, ‘How did you hear about the WAI?’, ‘Have you ever used the WAI?’, ‘How have you used the WAI?’ and ‘How have the WAI results been used?’ The full questionnaire is available from the corresponding author upon request. The questionnaire was hosted on an online survey facility. UK and Finnish OH practitioners (broadly defined) were invited to complete the survey over a 2-month period in 2012. The current analysis is restricted to responses from the two primary respondent groups: OH nurses and occupational physicians. A link to the questionnaire was issued by email to members of the Society of Occupational Medicine (SOM), the OH nursing register hosted by SOM, the Finnish Association of Occupational Health Nurses and the Finnish Association of Occupational Health Physicians. In addition, the JISCMAIL OH mailing list (OCC-HEALTH@JISCMAIL.AC.UK) was used. Ethical approval was granted by the Research Ethics Committee of the Institute of Work, Health, & Organisations at the University of Nottingham.

## Results

The questionnaire was completed by 436 OH nurses and physicians who worked exclusively or primarily in the UK and 97 who worked in Finland. Respondents’ demographics and occupational characteristics are presented in Table 1. The authors did not have access to membership information for the participating groups so it was not possible to calculate response rates.

**Table 1.** Respondent demographic and occupational characteristics

	United Kingdom, *n* (%)	Finland, *n* (%)
Age (years)		
20–29	2 (<1)	2 (2)
30–39	42 (10)	20 (20)
40–49	166 (38)	30 (31)
50–59	183 (42)	30 (31)
60+	37 (8)	15 (16)
Not specified	6 (1)	–
Gender		
Male	98 (22)	26 (27)
Female	335 (77)	71 (73)
Not specified	3 (1)	
Occupation		
OH nurse	336 (77)	50 (52)
OH physician	100 (23)	47 (48)

Data is presented in the form of frequencies. Two items involved open-ended responses; these concerned understanding of WA and factors influencing use of the WAI in Finland. Thematic analysis procedures were used to group responses into categorical themes [15].

All Finnish respondents and 96% of UK respondents were aware of the term ‘work ability’. Respondents reported diverse understanding of the term (Table 2). Among the UK sample, WA was primarily understood to be an indicator of ability to work, while health and work balance was the most common response from the Finnish sample.

**Table 2.** Respondents’ understanding of the term ‘work ability’

	Finland, *n* (%)	United Kingdom, *n* (%)
Health and work balance	26 (28)	75 (20)
Biopsychosocial	18 (19)	9 (2)
Fitness to work	17 (18)	67 (18)
Health and life balance	11 (12)	–
Ability to work	11 (12)	144 (38)
Well-being	7 (7)	1 (<1)
Performance standards	4 (4)	22 (6)
Functional assessment	–	64 (17)

There was disparity between Finnish and UK respondents regarding knowledge of the WAI: 39% (UK) and 94% (Finland) had heard of the instrument. Among the UK sample, the majority of respondents aware of the WAI had heard about it from a peer-reviewed journal (49%). This was followed by OH and/or safety colleagues (34%), other journals/magazines (32%), conference/lecture (20%), having seen it in use (6%), training course (5%) and own research (5%). Among the Finnish sample, the most frequent source of knowledge of the WAI was having seen it in use (92%). This was followed by OH and/or safety colleagues (38%), training course (31%), conference/lecture (30%), other journals/magazines (16%), peer-reviewed journal (15%) and own research (15%). Approximately half (48%) of UK respondents attributed failure to use the WAI in OH practice to a lack of training.

Ninety-five per cent of Finnish and 7% of UK respondents reported that they had used the WAI. Both UK and Finnish respondents within these subgroups indicated that they primarily used it for individual case-management activities. However, Finnish respondents also reported applications in workplace health promotion activities, health surveillance, as an element of an ageing workforce management programme and as a research tool.

Finnish respondents indicated that WAI scores were used to understand individual health trends (86%), understand working population health trends (70%), understand WA of all ages (71%), contribute towards health promotion and well-being initiatives (64%), promote rehabilitation back to work (58%), predict sickness absence (46%), understand the WA of older employees (33%), conduct WA research (24%) and understand the WA of younger employees (13%).

Factors reported by Finnish respondents as influencing use of the WAI in OH practice included the following (in descending order): its use being common practice; offering an effective system for individual health checks; and providing opportunities for organizational trend analyses, organizational policy and procedure, future workforce planning, rehabilitation, disability assessment and health promotion. Two per cent of the Finnish sample indicated they did not like using the WAI in their practice.

**Figure 1.**
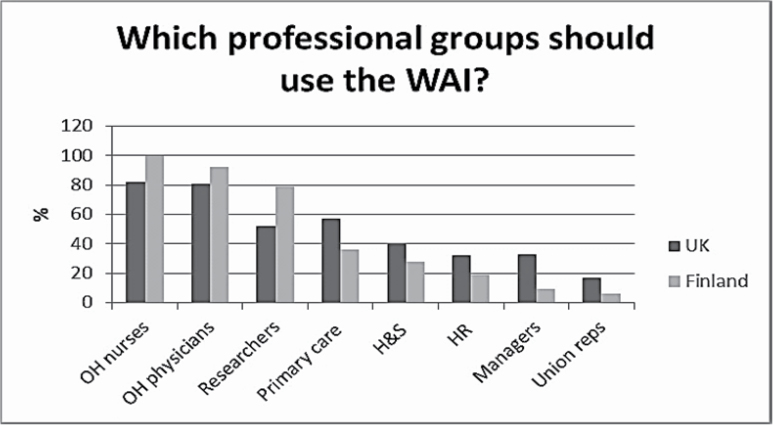
Respondents’ views on who should be able to use the WAI.

Respondents’ views on the professional groups that ought to use the WAI are presented in Figure 1. Both Finnish and UK respondents identified OH nurses and physicians as the two main groups that ought to use the instrument. Finnish respondents identified researchers as a third eligible group; UK respondents suggested primary care practitioners.

Respondents were asked which version of the WAI they used. Only 13 UK participants responded to this question. Nevertheless, results indicated that all three versions were used: long version (39%) [16], short version (46%) [12], single-item version (15%) [17]. Among the Finnish sample (*n* = 83), a preference was expressed for the long version (63%), followed by the short (35%) and single-item (3%) versions. Thirteen Finnish respondents indicated that they use all three versions in their practice.

## Discussion

Our study found that knowledge and understanding of the WA concept was high and use of the WAI was viewed as common practice among responding Finnish OH professionals; in contrast, only 39% of UK respondents knew of the WAI and even fewer (7%) had used it. Lack of training was viewed by UK respondents as a barrier to the expanded use of WA and the WAI in UK OH practice. Our study is the first of its kind to examine OH practitioners’ knowledge, understanding and use of WA and the WAI in their professional activities.

The study was limited by the fact that it was not possible to sample from the entire population of OH nurses and physicians in Finland and the UK. Hence the views expressed may not reflect those of these populations as a whole. It is also possible that the exclusive use of English in the questionnaire may have presented a barrier to completion for some Finnish respondents. Further, it is possible that self-report bias may have produced an idealized view of practice [18].

This study did not seek to identify possible factors underpinning disparities between UK and Finnish respondents in understanding and applying WA. Future research might usefully explore whether the differences identified reflect contrasting OH systems and practice in these countries. Indeed the promotion of WA is enshrined in the Finnish Occupational Health Act of 2002 [19] and the Occupational Safety Act of 2003, which state that ‘Maintaining work ability during aging is our common goal’ [20]. No such provisions exist in UK legislation. Furthermore, Finnish employers are required to provide an OH service [19]; in the UK, where no such requirement exists, it is estimated that up to 70% of workers have no access to OH services [21]. As such, opportunities for UK-based OH practitioners to use WA are fewer.

The study found that all three versions of the WAI were utilized by OH practitioners in Finland and the UK. Among UK respondents, the short version was the most commonly used; among Finnish respondents, the long version. Both samples reported that the single-item version was rarely used. This is perhaps surprising given the benefits associated with use of brief questionnaires, including minimizing interruption to organizational activities, reduced cost, promotion of a strong response rate [22,23] and ease of interpretation. Research is warranted to further establish the reliability and validity of the short and single-item versions of the WAI in a variety of occupational and national contexts. There is evidence to suggest that UK OH physicians cite the primary reasons for failing to use questionnaires in their practice as lack of availability, insufficient time, negative attitudes towards questionnaires, insufficient evidence base and lack of endorsement by the Faculty of Occupational Medicine [24]. The existence of extensively validated short and single-item versions might generate further opportunities for OH practitioners to use WA in their activities, particularly in the UK where the concept is at present relatively novel and untested. Workplace interventions, to improve WA, have so far failed to demonstrate significant benefits [25], suggesting that further research in this regard is also required for WA to be accepted more widely. In addition, it is suggested that economic evaluation will increasingly play a role in decisions about OH interventions [26]. With these research needs in mind, the authors are currently engaged in a large-scale study examining WA in the UK manufacturing sector with a view to contributing to the evidence base to support the application of WA and the WAI in UK OH practice.

**Key points**

- The assessment of work ability is common in Finnish occupational health practice but not in the UK.
- UK occupational health practitioners report knowledge, understanding and use of the Work Ability Index that is substantially different to that of those in Finland.
- UK occupational health practitioners cite lack of available training as the primary barrier to the application of work ability in their practice.

## Conflicts of interest

None declared.
